# Implications of petrochemical exposure and epigenetic alterations on human health

**DOI:** 10.3389/ftox.2025.1542871

**Published:** 2025-03-13

**Authors:** Selvaraj Jayaraman, Anupriya Eswaran, Vishnu Priya Veeraraghavan, Mohammed Fazal, Adham Al-Rahbi, Srinivasa Rao Sirasanagandla

**Affiliations:** ^1^ Centre of Molecular Medicine and Diagnostics (COMManD), Department of Biochemistry, Saveetha Dental College and Hospitals, Saveetha Institute of Medical and Technical Sciences, Saveetha University, Chennai, India; ^2^ Department of Human and Clinical Anatomy, College of Medicine and Health Sciences, Sultan Qaboos University, Muscat, Oman

**Keywords:** petrochemicals, epigenetic modifications, methylation, radio-omics, therapeutic targets, hazardous exposure

## Abstract

The petrochemical industry and automobiles contribute significantly to hazardous waste, which contains a broad array of organic and inorganic compounds posing serious health risks. Identifying biomarkers of exposure and creating predictive models for toxicity characterization necessitate a thorough understanding of the underlying epigenetic mechanisms. The development of disease is intricately linked to epigenetic processes, such as DNA methylation, histone modifications, and microRNA (mi-RNA) regulation, which mediate gene-environment interactions. While previous studies have investigated these alterations as markers for petrochemical-induced changes, there is still a need for deeper exploration in this area, with particular emphasis on advanced gene-editing technologies. This review highlights the specific epigenetic processes, especially gene-specific DNA methylation changes, associated with prolonged petrochemical exposure. Notably, the demethylation of long interspersed nuclear element 1 (LINE-1), Alu elements, and forkhead box P3 (FOXP3), as well as hypermethylation of interferon gamma (IFN-γ) and hypomethylation of interleukin-4 (IL-4) promoter regions, are discussed. These alterations in DNA methylation patterns serve as valuable biomarkers, potentially offering insights into early detection and personalized treatment options for diseases caused by long-term exposure to petrochemicals. Furthermore, CRISPR-based gene editing techniques, while underexplored, present a promising approach for correcting petrochemical-induced mutations. In addition, AI-driven radiomics holds promise for early disease detection, though it is currently limited by its lack of integration with multi-omics data. In conclusion, it is crucial to refine disease modelling, develop comprehensive risk assessment models, and innovate targeted therapeutic strategies. Future research should focus on enhancing exposure evaluation, incorporating computational tools to analyze molecular changes, and improving our understanding of how these modifications influence disease prevention and treatment.

## Graphical Abstract

**Figure:**
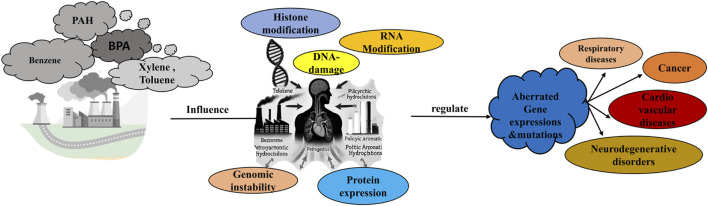
The graphical abstract illustrates the impact of petrochemical pollutants such as polycyclic aromatic hydrocarbons (PAHs), benzene, xylene, toluene, and bisphenol A (BPA) on epigenetic mechanisms. These chemicals induce histone modifications, DNA damage, RNA modifications, and genomic instability, leading to aberrant gene expressions and protein alterations. Such epigenetic dysregulation is associated with the onset of diseases including cancer, respiratory illnesses, cardiovascular diseases, and neurodegenerative disorders.

## 1 Introduction

The petrochemical industry, which produces compounds derived from petroleum and natural gas, is essential to the global economy due to its extensive applications across multiple sectors, such as detergents, cosmetics, food, agriculture, and dyes (Tripathy et al., 2017). However, the rapid expansion of this industry has led to significant environmental concerns due to the release of hazardous waste. These wastes include organic pollutants, such as benzene, toluene, ethyl benzene, and xylene (BTEX), and inorganic compounds, including heavy metals like lead, cadmium, and mercury (Zhen et al., 2017). The industry also produces solid waste, such as carbon black, contaminated sludge, and effluents containing polycyclic aromatic hydrocarbons (PAHs) (Fulekar and Pathak, 2024). These substances persist in the environment, contaminating soil, water, and air, and causing long-term ecological damage (Prüss-Ustün et al., 2016) Humans who are exposed to petrochemical pollution may experience serious health issues primarily through epigenetic modifications, which involve heritable changes in gene expression without altering the DNA sequence itself (Dolinoy et al., 2007). For example, exposure to benzene, a common component of petrochemicals, can disrupts gene function and increases cancer risk (McHale et al., 2012). Another important class of petrochemical pollutants that are known to cause oxidative stress, inflammation, and DNA damage is polycyclic aromatic hydrocarbons (PAHs).

These effects further lead to the development of numerous diseases, including cancer. The atmosphere is exposed to these persistent organic pollutants due to incomplete fossil fuel combustion, industrial activities, and automobile emissions. When PAHs enter the human body, they are broken down into reactive intermediates that can bind to DNA and cause mutagenesis and cancer. Long-term exposure to PAHs has been connected to respiratory and cardiovascular conditions, as well as skin, bladder, and lung malignancies (Ho et al., 2012). The toxic effects of these pollutants are exacerbated by their ability to interfere with key biological processes, including DNA repair, apoptosis, and immune responses (Chung and Herceg, 2020).

Exposure to petrochemical pollutants has been linked to a wide range of diseases, including cancers, respiratory illnesses, cardiovascular diseases, and neurological disorders. According to the World Health Organization (WHO), around 20% of all cancers and approximately 31% of cardiovascular diseases globally are attributed to environmental pollutants, including those from petrochemicals (Prüss-Ustün et al., 2016). Petrochemicals such as benzene are well-documented causes of haematological malignancies like acute myeloid leukemia, particularly in occupational settings like petroleum refineries and chemical plants (McHale et al., 2012). Moreover, PAHs have been shown significant increase in the risk of lung cancer and other respiratory conditions by forming DNA adducts and inducing mutations (Piskonen and Itävaara, 2004).

Numerous epigenetic changes, such as DNA methylation, histone modifications, and shifts in microRNA (miRNA) expression, have been connected to petrochemical exposure. These changes are linked to higher risks of diseases like cancer, reproductive disorders, metabolic problems, and neurodevelopmental outcomes (Kubota et al., 2012). They can also interfere with the expression of certain genes. In order to reverse the effects of petrochemical-induced mutations and restore normal gene function, advances in CRISPR-based gene editing have made it possible to precisely modify epigenetic regulators Additionally, it has been determined that petrochemical-induced mutations in important oncogenes and tumor suppressor genes could be targets for epigenome editing as an early disease intervention strategy (Kim et al., 2012; Zhao et al., 2023).

The detection of diseases associated with petrochemical exposure—which is linked to cancer, heart disease, and respiratory disorders because of harmful substances like benzene and PAHs—is being revolutionized by AI and radio-omics (Tunali et al., 2021). Radio-omics uses CT, MRI, and PET scans to extract quantitative imaging features, and AI-driven models improve analysis by spotting disease signatures like cancer, COPD, and lung fibrosis for early diagnosis (Gillies et al., 2016; Maniaci et al., 2024). For instance, hepatic fibrosis and renal toxicity can also be detected with the help of AI-powered radio-omics (Chassagnon et al., 2020). Beyond environmental health, AI-integrated radio-omics enhances personalized treatment and diagnostic precision, particularly in oncology (Lambin et al., 2017). Better disease monitoring and risk prediction could result from its integration into occupational health, despite obstacles like data standardization. Since, exposure to petrochemicals poses a serious public health risk, underscoring the necessity of efficient interventions is needed.

Even though the toxicological effects have been extensively studied, little is known about the underlying epigenetic mechanisms, particularly in cases of prolonged exposure. The purpose of this review is to investigate the ways in which petrochemicals cause particular epigenetic changes that may function as exposure biomarkers and targets for treatment. CRISPR-based gene editing for reducing petrochemical-induced mutations has not received much attention. Although AI-driven radiomics lacks integration with multi-omics data, it has potential for early disease detection. For populations exposed over an extended period of time, risk models and targeted treatments are also required. In summary, this review aims to suggest innovative strategies to lessen the negative consequences of exposure to petrochemicals.

## 2 Overview on petrochemicals

Petrochemicals, primarily from crude oil and natural gas, are vital in producing various products like plastics and pharmaceuticals. While the industries significantly boost the global economy, it also possesses health and environmental risks due to the toxic nature of these compounds (Hsu and Robinson, 2024). They are broadly classified into three categories: Olefins, Aromatics, and Synthesis gas (syngas). Olefins, such as ethylene, propylene, and butadiene, are mainly used in the manufacturing of plastics, synthetic fibres, and elastomers (Zhang Y. et al., 2024). Benzene, Toluene, Xylene, which are basically aromatics are used as precursors in the production of dyes, detergents, and solvents. Syngas, a mixture of carbon monoxide and hydrogen, is used in producing ammonia for fertilizers and methanol for various chemical syntheses. Although conventional petrochemicals continue to dominate the market, there has been a shift in the last 10 years towards the manufacture of petrochemicals with reduced environmental footprints, such as bio-based alternatives (Zhang X. et al., 2024).

Olefins like ethylene and propylene are vital in the petrochemical industry but come with notable environmental and health risks. Their high reactivity contributes to ground-level ozone formation, a key component of smog, which can cause respiratory problems. The production process for olefins, often reliant on fossil fuels, is energy-intensive and generates significant carbon emissions, exacerbating climate change (Phillips, 1999). Additionally, ethylene oxide, a derivative of ethylene, is a known carcinogen linked to cancers such as Leukemia. Environmental contamination from olefins, particularly through spills or improper disposal, can lead to ecosystem degradation (Phillips, 1999).

The persistence, toxicity, and bioaccumulation of aromatic chemicals possess a serious risk to human health and the environment. The main ways that these substances enter the environment are through incomplete combustion of fossil fuels, organic materials, and different industrial processes. Common aromatic hydrocarbon benzene is utilized mostly as a solvent in the chemical industry and can be found in gasoline and crude oil. Benzene exposure reported to be associated with the development of acute myeloid leukemia and other hematological malignancies (McHale et al., 2012).

One of the types of PAH called benzo[a]pyrene (BaP) is a strong carcinogen that is created when organic material burns incompletely. It poses significant health risks, including lungs, skin, and bladder cancers, due to its tendency to bioaccumulate in fat tissues (Phillips, 1999). Aromatic hydrocarbons that are polycyclic (PAHs) PAHs are recognized for their carcinogenic qualities and are composed of two or more fused benzene rings. They are released into the environment during fossil fuel combustion and through petroleum leaks, leading to soil and water contamination (Fulekar and Pathak, 2024; Piskonen and Itävaara, 2004). Once present in the air, soil, or water, PAHs can remain for long periods, leading to chronic environmental contamination. Exposure to these substances is associated with serious health risks, including respiratory issues, immune system impairment, and an elevated risk of cancer, particularly in populations living in urban and industrialized areas. The significant environmental and health challenges posed by aromatic compounds highlight the urgent need for stricter regulations and continued research to control their release and minimize their impact on ecosystems and public health (Kim et al., 2013). Syngas production, while crucial for industrial processes, has significant environmental drawbacks due to the emission of greenhouse gases and pollutants. This impact is particularly severe when fossil fuels are used, contributing to air pollution and climate change. Even biomass-based syngas can lead to harmful emissions if not managed properly. To address these challenges, there is a focus on developing cleaner technologies and incorporating carbon capture and storage to minimize the environmental footprint.

Syngas, also known as synthesis gas, is a combination that mostly consists of carbon dioxide (CO_2_), hydrogen (H_2_), and carbon monoxide (CO). It is produced through processes like gasification, where carbon-containing materials such as coal, natural gas, or biomass are converted into syngas by reacting them with oxygen or steam (Woolcock and Brown, 2013). Syngas is versatile, serving as a key intermediary in the production of fuels like synthetic diesel and chemicals such as methanol and ammonia. There are different types of syngas which include Coal-based syngas which are generated from coal gasification, it is often associated with high levels of pollutants like sulphur and mercury, along with significant CO_2_ emissions. Natural gas-based syngas tends to be produced through steam methane reforming, and this is cleaner than coal-based but still contributes to greenhouse gas emissions. Biomass-based syngas tend to be created from organic materials like wood, agricultural waste, or even municipal waste. This type is considered more sustainable but can still produce harmful emissions if not managed correctly (Roseno et al., 2018). The production of syngas poses serious environmental and health risks. The release of greenhouse gases like CO_2_ from syngas production processes contributes to climate change, leading to extreme weather events, rising sea levels, and health issues related to air pollution. Additionally, the toxic components in syngas, such as carbon monoxide, can pose direct risks to human health, particularly in poorly ventilated areas where it might accumulate. Addressing these threats requires the adoption of cleaner production methods and technologies to reduce emissions and manage the pollutants associated with syngas production (Woolcock and Brown, 2013; Roseno et al., 2018; Johnston and Cushing, 2020; Gao et al., 2023).

### 2.1 Other petrochemicals

Additional petrochemicals, such as toluene, xylene, ethylene, and styrene, are used in solvents, paints, and plastics. Chronic exposure to these compounds can lead to neurotoxicity, liver and kidney damage, and other systemic health issues. They are made from natural gas and crude oil, are essential to the manufacturing of many common place items, such as paints, plastics, and solvents. Toluene, xylene, ethylene, and styrene are some of the most widely utilized petrochemicals; each has a specific role in industrial settings (Sharma et al., 2017). The main applications for toluene are as a solvent in adhesives, paint thinners, and paints. It is also used as a precursor in the synthesis of other compounds, including benzene. Prolonged exposure to toluene can cause neurotoxicity, which can include headaches, light headedness, and cognitive decline. Prolonged exposure can harm the liver and kidneys, making it harder for them to remove waste from the body (Abadin et al., 2007).

Further, Xylene, a solvent commonly used in the printing, rubber, and leather industries, consists of ortho-, meta-, and para-isomers despite having effective benefits its chronic exposure poses significant health risks. Its prolonged contact has been linked to neurological issues such as dizziness, confusion, and impaired motor skills, along with respiratory problems, liver and kidney damage. Recent studies also indicate potential cancer risk and concerns about xylene’s role in endocrine disruption, underscoring the need for deeper evaluations of its long-term health impacts (Goldstein, 1977; Rajan et al., 2014).

Polyethylene, one of the most extensively used polymers, is made in large part from ethylene. Extended exposure to ethylene can cause respiratory issues and skin irritation, even though it is not as hazardous as toluene and xylene. Long term exposure of ethylene has been proven to be a carcinogenic agent (International Agency for Research on Cancer. Some industrial chemicals, 1994; Kirman et al., 2021). Styrene is used to create polystyrene plastics and resins. Chronic styrene exposure has been associated with several health hazards, including neurotoxicity, skin irritation, and respiratory problems. Additional research is needed to establish definitive links between styrene exposure and an increased risk of cancer. Despite these petrochemicals are essential to many different sectors, long-term exposure can have detrimental effects on the liver, kidneys, and nervous system, among other organ systems. Safeguards and restrictions must be put into place in order to reduce hazardous exposures (Atsdr, 1997; Sadighara et al., 2022).

## 3 Mechanism of petrochemical-induced health risks

**FIGURE 1:**
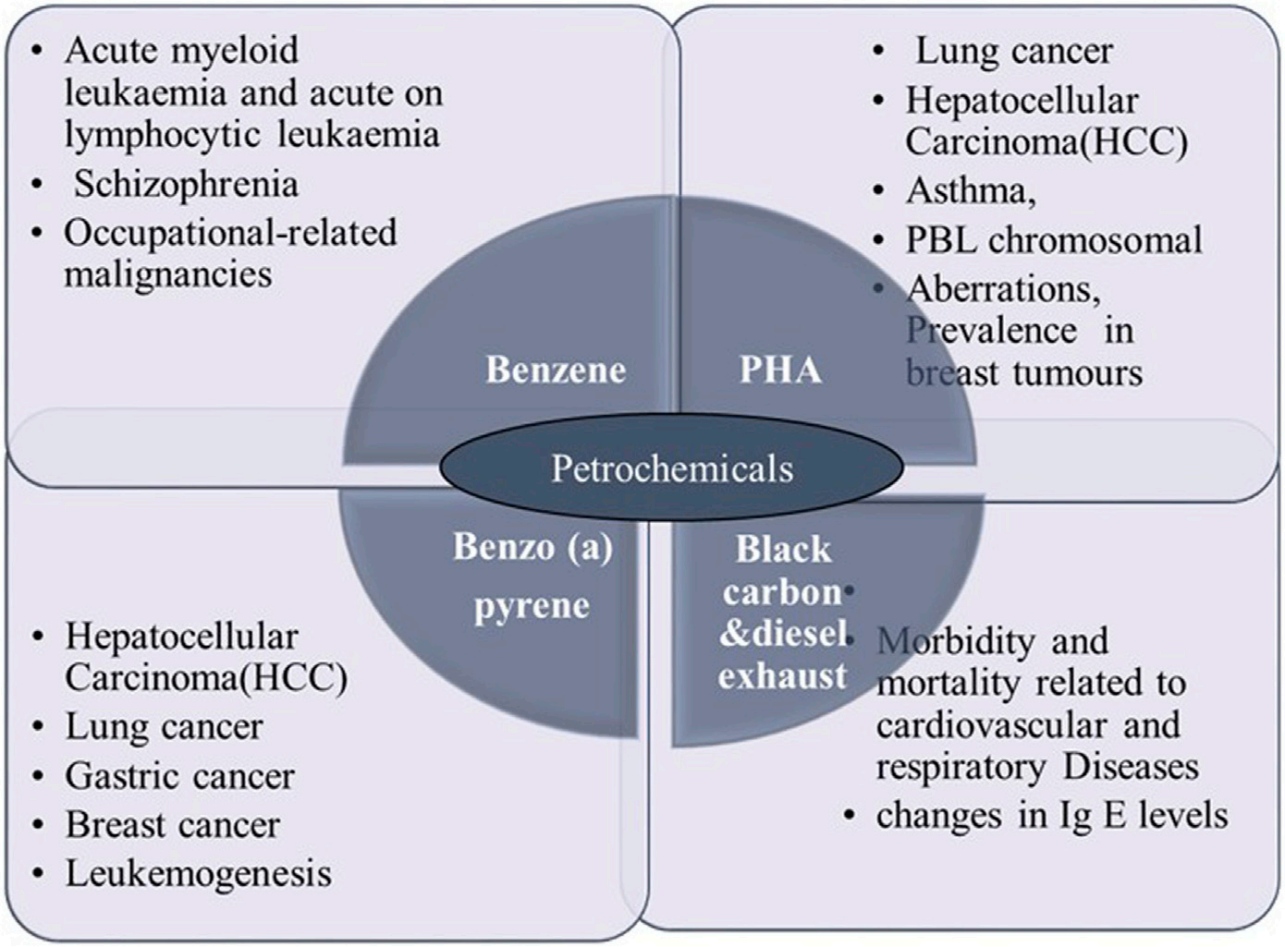
Petrochemicals exposure and their diseased risks. This figure illustrates the major petrochemicals—Benzene, Polycyclic Hydrocarbons (PHA), Benzo(a)pyrene, Black Carbon, and Diesel Exhaust Particles—along with their corresponding health effects. Benzene is linked to conditions such as acute myeloid leukemia, schizophrenia, and occupational-related malignancies. PHA exposure is associated with lung cancer, hepatocellular carcinoma (HCC), asthma, and chromosomal aberrations in breast tumors. Benzo(a)pyrene is linked to HCC, lung cancer, gastric cancer, breast cancer, and leukemogenesis. Black Carbon and Diesel Exhaust Particles are known for contributing to cardiovascular and respiratory diseases, including changes in immunoglobulin E (IgE) levels.

Petroleum hydrocarbons are characterized by their high boiling points and varying molecular weights, leading to diverse toxic effects on the environment and human and animal health. The toxicity of PHs depends on factors such as susceptibility, exposure duration, concentration, and chemical composition. They can impact multiple human systems, including the neurological, circulatory, immunological, and endocrine systems, and contribute to metabolic and hormonal problems. PH’s exposure has been linked to mutagenicity, genotoxicity, carcinogenicity, and various organ toxicities, including hepatotoxicity and nephrotoxicity (Vandana et al., 2022). These can contact the skin, be ingested, or be inhaled by humans. When these substances are metabolically activated within the body, reactive intermediates are created. These intermediates can then attach to proteins, DNA, and RNA in cells, causing oxidative stress and cellular damage. Petrochemicals release volatile organic compounds (VOCs) and polycyclic aromatic hydrocarbons (PAHs) during manufacturing, transportation, and use, which pose the greatest health hazards (Figure 1). These compounds can have a variety of toxicological effects when they enter the human body through ingestion, inhalation, or skin contact. Mechanistically, petrochemicals can disrupt cellular processes by inducing oxidative stress, altering gene expression, and damaging cellular membranes. Long-term exposure to certain petrochemicals, such as benzene and formaldehyde, has been linked to the development of malignancies, including leukaemia and lymphoma, due to their ability to cause DNA mutations and impair the body’s repair mechanisms (Johnson et al., 2010; Lawal, 2017).

### 3.1 Health risks: acute and chronic exposure

Petrochemical exposure is linked to a multitude of health issues, and the consequences can be either acute or chronic, contingent on exposure duration and quantity. Acute exposure can cause symptoms like headaches, vertigo, breathing problems, and irritation of the skin and eyes right once, especially when exposed to high concentrations of petrochemical pollutants. These symptoms are frequently the body’s quick reaction to the harmful effects of volatile chemicals like benzene, toluene, and xylene. For instance, breathing in these substances may irritate the central nervous system and respiratory tract, resulting in lightheadedness and respiratory discomfort (Wilson et al., 2021). However, chronic exposure to lower petrochemical concentrations tends to have more subtle and serious health impacts that develop over months or years. A concerning consequence of extended exposure is the increased likelihood of getting several types of cancer.

Common petrochemical benzene is a known carcinogen that has a high correlation to hematological malignancies like lymphoma and leukemia. Constant exposure causes abnormal cell function to be disrupted, which results in mutations and unchecked cell growth that eventually leads to cancer (Smith, 2010). Moreover, exposure to petrochemicals has been associated not only with cancer but also with neurological diseases. For example, it has been demonstrated that the compound styrene, which is commonly used in the manufacturing of plastics and synthetic rubber, causes neurological abnormalities (Abadin et al., 2007). As stated, extended exposure to styrene can cause peripheral nervous system damage, cognitive difficulties, and memory problems. These consequences result from the accumulation of styrene and related compounds in the fatty brain tissues, which can cause harmful interference with the transmission of nerve signals (Abadin et al., 2007).

Furthermore, people who are exposed to petrochemicals for extended periods of time should be concerned about reproductive damage and endocrine disruption. Plasticizers such as phthalates can disrupt the balance of hormones, affecting both prenatal growth and fertility (Smith, 2010). Generally, Children born to women who gets exposed to these substances during their pregnancy may experience developmental delays, poor fertility, and birth deformities because of chronic exposure. Furthermore, metabolic abnormalities and an elevated risk of diseases like diabetes and obesity can result from petrochemicals' disruption of endocrine function. To reduce these hazards, strict laws and public health initiatives are necessary, as evidenced by both acute and chronic exposure.

## 4 Epidemiology of petrochemical exposure

Numerous harmful health effects have been linked to exposure to petrochemicals, as demonstrated by extensive epidemiological research. When compared to the general population, communities living close to petrochemical plants and those employed in the manufacturing or processing of petrochemical products are disproportionately affected by chronic health issues like cancer, respiratory disorders, and cardiovascular diseases (Landrigan et al., 2018). One major contributing factor to the development of cancer is thought to be prolonged exposure to harmful chemicals emitted during petrochemical processes, such as benzene, volatile organic compounds (VOCs), and other carcinogenic substances (Landrigan et al., 2018).

In a comprehensive meta-analysis spanning Europe, North America, and Asia, researchers discovered a persistent correlation between exposure to petrochemicals and increased mortality rates, specifically from respiratory and cardiovascular ailments. The results of this meta-analysis demonstrated that extended exposure causes chronic illnesses and speeds up the course of those illnesses, which can result in early mortality (Lee et al., 2022). The impacts of environmental stressors are amplified in places with poor air quality due to pollution from petrochemical industries. Furthermore, exposure to petrochemicals can have detrimental impacts on specific populations. Due to their still-developing immune and respiratory systems, children are more vulnerable to long-term harm (James et al., 2020). In a similar vein, elderly people are more vulnerable to worsening respiratory or cardiovascular conditions because they may already have weakened health.

Exposure to petrochemical pollutants can exacerbate the symptoms of pre-existing diseases, including asthma, cardiovascular disease, and chronic obstructive pulmonary disease (COPD) and hence, increasing the risk for these individuals (James et al., 2020). Public health experts stress the significance of strict regulatory measures to regulate petrochemical emissions and reduce human exposure in light of these findings. Mitigating the health risks associated with petrochemical exposure requires implementing strategies including air quality monitoring, enforcing industrial safety requirements, and guaranteeing the use of protective equipment for workers in petrochemical companies (Lee et al., 2022).

### 4.1 Prevention of petrochemical health risks

There are several health hazards associated with petrochemical exposure, such as cancer, neurological damage, non-communicable diseases and respiratory disorders. These compounds include benzene, toluene, and xylene. Personal protection equipment (PPE) must be used in the workplace to reduce the risks of inhalation and skin contact with hazardous chemicals (Deziel et al., 2015). Permissible exposure limits are enforced by regulatory frameworks, including those set by the Occupational Safety and Health Administration (OSHA), to reduce occupational dangers (Sørensen et al., 2005). Environmental regulations that limit the discharge of petrochemical pollutants into the air and water are essential for lowering the hazards to human health on a larger scale (Mohan and Ethirajan, 2012). Furthermore, to lessen the negative effects of petrochemical spills and contamination on human health, innovations such as air purification systems and soil bioremediation methods, which employ microbial degradation, are essential (Zhang et al., 2019). Reducing health risks in daily life also requires public education on the proper use and disposal of petrochemicals (Cutchin et al., 2008).

## 5 Epigenetic mechanisms

Environmental toxicants, such as petrochemical residues, can modify epigenetic mechanisms, including DNA methylation, histone modifications, and miRNA expression. These changes, influenced by factors like toxins, age, gender, and lifestyle, can disrupt genome function and contribute to various diseases (Marsit, 2015). DNA methylation, a key epigenetic marker, involves the addition of a methyl group to cytosine at CpG dinucleotides, often found in CpG islands within gene promoters. In normal cells, these regions remain unmethylated, whereas CpG hypermethylation silences genes, and global hypomethylation leads to genomic instability, both contributing to disease (Fenga et al., 2016). It is an epigenetic modification that regulates gene expression without altering DNA sequence. It occurs during cell division and is heritable, often repressing transcription by preventing RNA polymerase recognition. Methylation also influences chromatin structure by regulating enzymes like histone deacetylase. Aberrant DNA methylation is linked to diseases such as imprinting disorders, cardiovascular and autoimmune diseases, neurological disorders, and cancer. It also plays a role in memory storage, aging estimation, and biological clocks. DNA demethylation occurs through the base-excision repair system (Fenga et al., 2016; Feil and Fraga, 2012). Histone modifications, including methylation, acetylation, phosphorylation, ADP-ribosylation, and ubiquitylation of histone tails, play a crucial role in regulating chromatin structure and gene expression. Acetylation generally promotes transcription, while methylation can either activate or repress transcription depending on the lysine position involved (Fenga et al., 2016). These modifications are heritable changes in gene expression that occur without altering the DNA sequence and are part of broader epigenetic mechanisms, which also include non-coding RNAs and chromatin alterations like acetylation and methylation (Feil and Fraga, 2012). Chromatin, beyond its role in DNA packaging and mitosis, is central to gene regulation (Yan and Boyd, 2006). The nucleosome, composed of 147 base pairs of DNA wrapped around histones (H2A, H2B, H3, and H4), undergoes modifications that influence gene accessibility (Fabbri et al., 2007; Garzon et al., 2009). Increased acetylation activates transcription, while decreased acetylation leads to gene repression. Furthermore, these epigenetic changes are sensitive to environmental factors, with recent research emphasizing their role in cancer development, such as in pancreatic ductal adenocarcinoma (PDAC) (Chen et al., 2024). miRNAs, small non-coding RNAs, regulate gene expression post-transcriptionally by binding target mRNAs for inactivation. They play critical roles in cell functions such as proliferation, apoptosis, and genome stability. miRNAs and epigenetic modifications interact bidirectionally-epigenetic mechanisms regulate miRNA expression, while miRNAs influence chromatin structure and gene silencing (Fenga et al., 2016). Exposure to petrochemicals alters miRNAs, which control gene expression and are implicated in cancer, heart disease, and neurotoxicity. MiRNA profiles are changed by pollutants such as PAHs and benzene, which encourage immunological dysfunction, inflammation, and oncogenesis. Such alterations in miRNA could be biomarkers for diseases caused by petrochemicals. For instance, eexposure to benzene causes leukemia by upregulating miR-221 and miR-222 (Fenga et al., 2016), whereas PAHs promote miR-21, which inhibits tumor suppressors such as PTEN. PAH exposure’s alteration of miR-126 and miR-155 results in endothelial dysfunction and atherosclerosis (Fenga et al., 2016; Moffitt et al., 2015; Kandi and Vadakedath, 2015; He et al., 2021). Thus, environmental exposures, such as petrochemicals, can significantly impact epigenetic modifications, affecting gene expression and disease risk (Bollati et al., 2010).

### 5.1 Petrochemicals and epigenetic alterations

The production of crude oil and/or natural gas, as well as its exploration and drilling, are among the many activities that make up the upstream petroleum industry, which is a major contributor to global energy (Economides and Nolte, 2000). Natural oil seeps, industrial waste products and emissions, oil storage wastes, unintentional spills from oil tankers, coal tar processing wastes, petrochemical industrial effluents and emissions, etc., are some of the ways that aromatic compounds enter the environment (Miedema et al., 2000; Sucker et al., 2008). There is substantial evidence linking occupational exposure to benzene, notably in petroleum refineries, to an increased incidence of hematological malignancies, including acute myeloid leukemia (Smith, 2010; Rinsky et al., 2002; Whitworth et al., 2008). According to *vitro* research, exposure to benzene causes the poly (ADP-ribose) polymerase-1 (PARP-1) gene, which is critical for DNA repair, to become hypermethylated. DNA methylation alterations like those seen in hematological malignancies have been linked to even low-dose airborne benzene exposure in healthy persons. These modifications include hypermethylation of the p15 tumor suppressor gene, hypomethylation of the MAGE1 (Melanoma antigen-1) element, and hypomethylation of the LINE-1 and Alu elements. Prior study demonstrated that Acute myeloid leukemia, Myelodysplastic syndrome and acute lymphoblastic leukemia frequently exhibit p15 hypermethylation, whereas and lymphoma exhibit p16 hypermethylation. In which, workers exposed to benzene had significantly lower levels of p15 mRNA with 63% exhibiting low expression in contrast to 38% of controls. Hence, reportedly benzene-induced gene silencing is further supported by a strong negative correlation between promoter methylation and p16 mRNA levels. Thus, in populations that are exposed, the inactivation of these tumor suppressors might increase the risk of cancer (Koutros et al., 2010; Waggoner et al., 2011; Xing et al., 2010). Further, report from another study demonstrated that global DNA methylation altered by benzene exposure, leads to MGMT and hMLH1 hypermethylation and LINE-1 and Alu hypomethylation. Reportedly and hMLH1 hypermethylation rates were more common in 141 exposed workers, LINE-1 methylation was significantly lower which may have an adverse effect on DNA repair. Moreover, benzene metabolite-induced hMLH1 hypermethylation was validated by *in vitro* experiments. With decreased exposure, longitudinal data revealed decreased hMLH1 methylation and increased LINE-1 methylation, indicating reversibility. These results emphasize how benzene contributes to epigenetic modifications associated with carcinogenesis (Ren et al., 2019; Ji et al., 2010). Moreover, in human lymphoblastoid cells, it has been demonstrated that benzene metabolites decrease global DNA methylation (Ji et al., 2010). Benzene can cause chromosomal translocations that result in chimeric oncoproteins and cytogenetic abnormalities such as aneuploidy, which can affect DNA methylation and gene expression. Mutations and epigenetic changes can impact critical genes, which are frequently examined in surrogate cells such as peripheral blood lymphocytes (McHale et al., 2012).

In addition to being well-known carcinogens, PAHs are pervasive environmental contaminants. They exert toxicity through metabolic activation into electrophiles, generating reactive oxygen species (ROS). The nucleotide excision repair (NER) pathway is primarily responsible for repairing PAH-induced DNA damage, indicating a potential NER-gene-environment interaction in PAH-associated carcinogenesis (Li et al., 2015). Benzo[a]pyrene (BaP) is a potent carcinogen which is frequently used as a biological tracer in studies investigating consequences on both human and animal health. BaP exposure has been shown to cause hypermethylation of tumor suppressor genes, silencing them in various cancers, including HIC1 in lung cancer (Peluso et al., 2012). Significant risks to human health and the environment are also associated with diesel emissions, which release pollutants such particulate matter (PM), hydrocarbons (HC), carbon dioxide (CO2), carbon monoxide (CO), and nitrogen oxides (NOx). Microarray profiling of human bronchial epithelial cells exposed to diesel exhaust revealed significant alterations in miRNA expression, with 197 out of 313 detected miRNAs being regulated by at least 1.5-fold (Jardim et al., 2009). It has been proposed by Kubota et al. that modifications to histones or DNA, such as methyl residues, are involved in epigenetic regulation. It is hypothesized that these modifications could regulate the epigenetic mechanism (Kubota et al., 2012).

Further, this study uses a multi-omics approach to evaluate single nucleotide polymorphism (SNP) profiles and DNA methylation in residents who have been exposed to multiple pollutants on a long-term basis. The results show that Aryl Hydrocarbon Receptor Repressor (AHRR) has hypomethylation due to PAH exposure, which is associated with oxidative stress and cancer risk. Additionally, certain genes (DFNA5, KIAA1199, LINC00673) and SNP’s (Single nucleotide polymorphism) (NFIC, NSF, RNF39) have differential methylation, which is linked to disease susceptibility. Polycyclic aromatic hydrocarbons (PAHs) and heavy metals both have an impact on epigenetic modifications, according to the study, which emphasizes the necessity of taking multi-pollutant exposures into account when evaluating health risks (Chen et al., 2024).

The alterations in epigenetic modifications reported in reaction to the disclosures surrounding petrochemicals may be causally linked to an increased susceptibility to illnesses (Table 1).

**TABLE 1:** Petrochemical-induced epigenetic modifications and their links to health disorders.

S.no	Petrochemicals	Epigenetics alternation	Associated health Conditions/Diseases
1	Benzene	DNA methylation Hypermethylation of the p15 tumour suppressor gene, global hypomethylation of the Alu and LINE-1 genes, P15 hypermethylation and (MAGE-1) hypomethylation, hypermethylation of the PARP-1 poly (ADP-ribose) polymerases-1, and histone modification	Acute myeloid leukaemia, schizophrenia and non-lymphocytic leukaemia (McHale et al., 2012; Smith, 2010; Fenga et al., 2016; Feil and Fraga, 2012; Yan and Boyd, 2006; Fabbri et al., 2007; Garzon et al., 2009; Shimabukuro et al., 2007)
		Global DNA methylation and promoter-specific methylation of the two tumor suppressor genes, p14*ARF* and p15*INK4b*	Occupational-related malignancies (Jamebozorgi et al., 2018)
		It promotes DNA repair mechanisms, but its metabolites inhibit Topo II, leading to DNA strands with adducts and resulting in double-strand breaks (DSBs). Excessive, unrepaired DNA damage triggers programmed cell death (apoptosis)	Causes Genomic instability, Haematological toxicity and Leukemogenesis (Dewi et al., 2020)
2	Polycyclic aromatic hydrocarbons (PAHs)	Demethylation of LINE1, Alu, and HERV, have been analyzed, in addition to specific genes including p53, p15, p16, APC, RASSF1A, HIC1, iNOS, hTERT, and IL-6	Changes in peripheral blood cells Lung cancer (Moffitt et al., 2015; Byun et al., 2013; Hou et al., 2014)
		Methylation of DNA (FOXP3, p14ARF, p15ink4b, and p16INK4a) and deregulation of miRNA	Hepatocellular Carcinoma (HCC) (Jacobs et al., 2017)
		Cord blood from newborns (WBCs) DNA methylation of the ACSL3 promoter, Intragenic p19INK4α DNA methylation in adult blood (PBLs), CDH1, HIN1, PARPβ, and TWIST promoter DNA methylation in adult tumor tissue DNA methylation of the ESR1 promoter, BRCA1, CCND2, and DAPK.	Childhood (≤5 years) Asthma, Adulthood PBL chromosomal Aberrations, Adulthood Prevalence in breast tumors (Ali and Wang, 2021)
	Polycyclic aromatic hydrocarbons (PAHs)	PAH leads to the formation of stable DNA adducts as their derivatives are metabolized in the body, causing oxidative DNA damage. Hydroxyl radicals (OH·) generated during inflammation further enhance nitric oxide production, which can modify cellular biomolecules. This damage affects DNA, proteins, and lipids, potentially resulting in genetic instability, apoptosis, and angiogenesis	If left unrepaired, these processes can lead to mutations, cancer development, cell death and hepatic Wilson disease (Ye and Xu, 2010)
3	Benzo[a]pyrene	Promoter DNA methylation	Risk of HCC (Jacobs et al., 2017)
		Methylation of Retinoic acid receptor beta2 (RAR-β2) gene promoter, Hypermethylation of HIC1	Lung cancer (Peluso et al., 2012; Ye and Xu, 2010)
		BaP induces cell damage and cancerization by forming DNA-adduct, oxidative stress. BaP targets DNA methyl transferases to induce DNA methylation, it can reduce genome-wide methylation levels and alter the methylation levels of specific genes, which can activate or inactivate tumor suppressor genes and lead to tumorigenesis. It also promotes hypermethylation, histone acetylation also hypomethylation and deacetylation in various cancer cells	Leads to increased inflammation and tumourogenesis particularly in breast, liver and gastric cancer cells (Wang et al., 2023a)
4	Diesel exhaust particles	Mice with hypomethylation at a CpG site of the IL-4 promoter and hypermethylation at multiple sites of the interferon gamma (IFN) promoter	Changes in IgE levels (Perera et al., 2009)
5	Black carbon	Methylation of Dna in repetitive Elements of LINE-1	Morbidity and mortality related to cardiovascular and respiratory diseases (Liu et al., 2008)

### 5.2 Perceptions of epigenetics towards petrochemicals

Identifying key genetic and epigenetic events in chemical carcinogenesis is crucial for discovering biomarkers that assess exposure, predict biological effects, and prevent adverse health outcomes (Ravegnini et al., 2015). In East Asian countries like Japan, epigenetic mechanisms have been a popular alternative medicine for over 2,100 years (Watanabe et al., 2008). Traditional Chinese medicine often includes substances that interact with epigenetic proteins, such as Polycomb group proteins, MBD (Methyl-CpG-binding domain), HDAC (Histone Deacetylase), and DNMT DNA (Cytosine-5)-Methyltransferase (Hsieh et al., 2011). These epigenetic modifications are a component of an intricate web of relationships that have the potential to reinforce one another and affect how genes are expressed. Comprehending these mechanisms could elucidate the ways in which environmental influences on the development of disease are influenced by DNA methylation, histone changes, and miRNAs (Fuks, 2005) (Table 2).

**TABLE 2:** Mechanism of epigenetic alternation in response to petrochemicals exposure.

S.no	Petrochemicals	Study type	Epigenetic mechanism	References
1	Benzene	*In vitro* and *In vivo*	Hypomethylation of repetitive elements in peripheral blood DNA from occupationally exposed individuals is linked to reduced MGMT promoter methylation, impairing DNA repair and increasing cancer susceptibility by hindering the repair of alkylated DNA.	Seow et al. (2012), Li et al. (2017)
		*In vitro*	Decreased levels of peripheral blood cells and bone marrow progenitor cells, along with dysregulated miRNAs like miR-34a-5p, miR-342-3p, and miR-100-5p, are linked to hematological malignancies	Wei et al. (2015)
		*In vitro*	Airborne benzene exposure is linked to reduced methylation of LINE-1 and AluI, with gene-specific effects like MAGE-1 hypomethylation and p15 hypermethylation, suggesting these epigenetic changes may contribute to benzene’s harmful health effects	Bai et al. (2014)
		*In vitro*	Benzene-exposed traffic policemen showed high mEH enzyme activity, predicted by exon 3 and 4 polymorphisms, which correlated with a lower frequency of micronuclei (MN), validated markers of genotoxicity formed during cell division due to chromatin or chromosome loss	Angelini et al. (2011)
		*In vitro*	At levels below the permissible threshold, long-term occupational benzene exposure can methylate tumor suppressor gene DNA, which could eventually result in the formation of cancer	Jamebozorgi et al. (2018)
2	Polycyclic aromatic hydrocarbons (PAHs)	*In vitro*	In workers exposed to polycyclic aromatic hydrocarbons (PAHs), LINE-1 and MGMT methylation levels were significantly reduced, with negative correlations between methylation and urinary 1-hydroxypyrene levels (r = −0.329, p < 0.001; r = −0.164, p = 0.049; r = −0.176, p = 0.034)	Li et al. (2017), Duan et al. (2013)
3	Benzo[a]pyrene	*In vitro*	BPDE exposure in esophageal cancer cell lines leads to methylation of the RAR-β2 gene promoter, with lung cancer linked to suppressed RAR-β2 expression in premalignant and malignant esophagus cells	(Ye and Xu, 2010)

**FIGURE 2:**
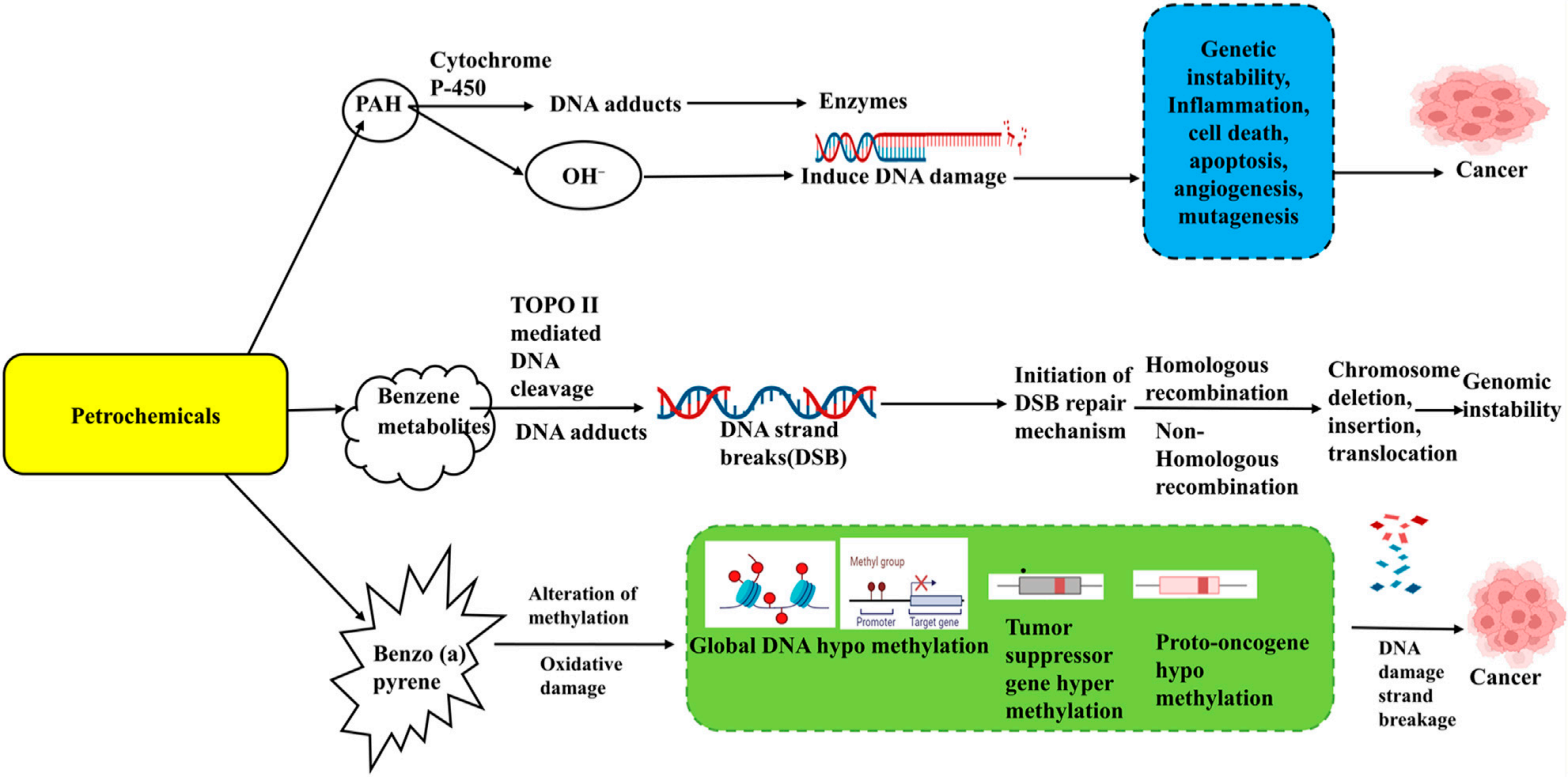
Impact of Petrochemicals on epigenetic modifications and disease progression. This figure illustrates the impact of petrochemicals on DNA methylation patterns and their associated epigenetic modifications. Petrochemicals such as benzene, polycyclic aromatic hydrocarbons (PAH), and Benzopyrene (BaP) can induce DNA strand damage and alterations in gene expression. These changes lead to global DNA hypomethylation, tumour suppressor gene hypermethylation, and proto-oncogene hypomethylation. Such epigenetic dysregulation results in genomic instability, inflammation, apoptosis, angiogenesis, and mutagenesis, eventually contributing to the development of various diseases, including cancer.

**FIGURE 3:**
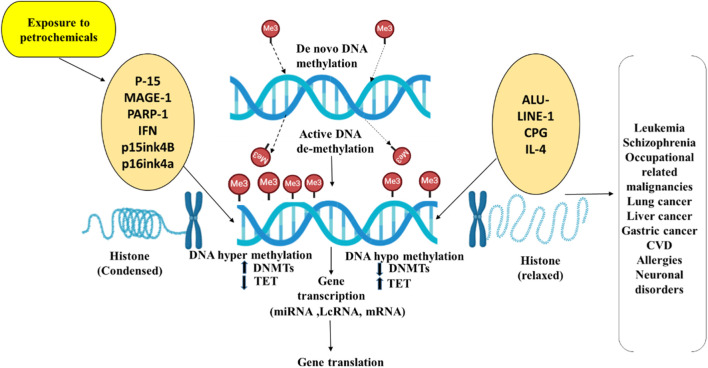
Connection between histone modifications and DNA methylation in gene regulation upon petrochemical exposure. The relationship between DNA methylation and histone modifications is depicted in this figure, emphasizing their functions in gene regulation and disease progression due to petrochemical exposure. Petrochemical exposure can lead to aberrant gene expression through either hypo-methylation (gene activation) or hyper-methylation (gene silencing), which in turn alters histone modifications. These epigenetic changes can contribute to disease conditions such as cancer, neurodegenerative disorders, and cardiovascular diseases (CVD).

Understanding the epigenetic mechanisms involved, benzene-related hypomethylation may serve as an intermediate link to its adverse health effects, offering insights for developing prevention strategies for exposed workers. MicroRNAs (miRNAs) could exhibit a crucial part in leukemogenesis and normal hematopoiesis and analyzing them may help identify new biomarkers for benzene toxicity. Recent studies reported differentially expressed miRNAs of account of 138 in the plasma of benzene-exposed workers (Lei et al., 2015; Mozzoni et al., 2023), with focal adhesion identified as the most enriched pathway, including targets like SOS2 (Son of Sevenless Homolog 2), VCL (Vinculin) and MAPK1 (Mitogen-Activated Protein Kinase 1). Another study on chronic benzene poisoning (CBP) patients found 13 significantly deregulated miRNAs, primarily associated with cancer pathways, targeting genes such as VEGFA (Vascular Endothelial Growth Factor A) and PTEN (Phosphatase and Tensin Homolog) (Bai et al., 2014; Mozzoni et al., 2023).Genetic susceptibility and polymorphisms in enzymes involved in PAH metabolism, such as CYP1A1 (Cytochrome P450 Family 1 Subfamily A Member 1) and GSTP1 (Glutathione S-Transferase Pi 1), may contribute to PAH-related toxicity. Furthermore, epigenetic changes through micro RNA deregulation and methylation of DNA on FOXP3, p14ARF can influence the final phenotype (Ravegnini et al., 2015). The Impact of Petrochemicals on epigenetic modifications and disease progression is given in (Figures 2, 3).

## 6 Petrochemicals on autophagy

Petrochemicals are a broad class of compounds that are produced from petroleum and natural gas. Examples of these chemicals are xylene, toluene, benzene, and PAH’s. Exposure of humans to these compounds is a serious problem because they are widely used in consumer products, industrial processes, and the environment. Research on these substances' influence on cellular functions, such as autophagy. Reactive oxygen species (ROS) are produced when oxidative stress is induced by certain petrochemicals, including benzene and PAHs. As a defensive mechanism to get rid of damaged cell parts and prevent more damage, this oxidative stress might trigger autophagy. On the other hand, high or sustained oxidative stress can overload the autophagic apparatus, impairing autophagy and ultimately causing cell death (Eldos et al., 2022).

Protective Mechanism: Autophagy serves as a crucial defensive mechanism in response to environmental stressors. Upon exposure to pollutants, the activation of autophagy may be enhanced to aid in the mitigation of cellular damage and the preservation of cellular integrity. Dual Role of Autophagy: Notably, autophagy may also assume a harmful role. In certain instances, exposure to pollutants can obstruct autophagic pathways or alter its function from a protective mechanism to one that promotes cellular apoptosis. This duality underscores the intricate nature of autophagy’s involvement in cellular responses to environmental stressors (Martínez-García and Mariño, 2020).

Benzo[b]fluoranthene precipitates podocyte damage predominantly via the suppression of autophagy. Autophagy constitutes a vital cellular mechanism that facilitates the degradation and repurposing of cellular constituents; its suppression may culminate in the aggregation of dysfunctional proteins and organelles, thereby ultimately leading to cellular impairment (Zhang et al., 2020). Trichloroethene (TCE) is a solvent widely employed in various industrial applications, and exposure to this chemical substance has been linked to an array of health issues, including inflammatory reactions and autoimmune conditions. The exposure to Trichloroethene (TCE) activates inflammatory signalling cascades, which are further aggravated by dysfunctional autophagic mechanisms. This disruption in regulatory mechanisms can lead to the aggregation of dysfunctional proteins and organelles, thereby further exacerbating inflammatory responses (Wang et al., 2023b).

BaP is a polycyclic aromatic hydrocarbon recognized for its carcinogenic attributes. It presents considerable health hazards, particularly in the gastrointestinal tract, it can interfere with cellular functionalities. BaP acts to impede the commencement of autophagy in intestinal porcine epithelial cells. Autophagy represents a cellular mechanism that facilitates the degradation and repurposing of cellular constituents, thereby playing a crucial role in the preservation of cellular homeostasis. The suppression of this mechanism by BaP may intensify cellular stress and contribute to the resultant apoptosis and disruption of tight junctions (Li et al., 2023). Benzene exposure is associated with an elevation in autophagic activity and a reduction in acetylation levels within the bone marrow mononuclear cells (BMMNCs) derived from individuals affected by this exposure. This observation implies that autophagy is a critical mechanism underlying the hematotoxic consequences attributed to benzene. Study highlighted that hydroquinone (HQ), a metabolic residue of benzene, is a potent enhancer of autophagy in BMMNCs and CD34^+^ progenitor cells. Importantly, this autophagic induction occurs independent of apoptotic processes, thereby revealing a distinct pathway through which benzene manifests its deleterious effects (Qian et al., 2019)

## 7 CRISP-R gene editing mechanism

CRISPR/Cas9 (Clustered Regularly Interspaced Short Palindromic Repeats) has transformed genetics by enabling precise, efficient, and cost-effective DNA modifications. This technology uses the Cas9 enzyme, guided by RNA, to target and edit specific genes, either by disrupting, deleting, or inserting genetic material (Xu, 2024).It has numerous uses in a variety of industries, including the development of pest-resistant crops, cancer treatment, genetic disorder therapies, and pharmaceutical drugs (Xu, 2024; Rager et al., 2019).A growing number of genetic diseases, such as α1-antitrypsin deficiency, hematopoietic disorders, Duchenne muscular dystrophy, hemophilia, and hearing loss, are being studied and treated using CRISPR-Cas9. It was previously difficult to modify the genome for hematologic disorders, but new research has demonstrated that CRISPR-Cas9 can fix genetic mutations in hematopoietic stem cells, opening the door for their application in transplantation treatments (Sharma et al., 2021). Further, cancer treatment has been transformed by CRISPR/Cas9, which allows for precise genome editing to target oncogenes and restore tumor suppressors like KRAS and TP53. Through T cell engineering in CAR-T cell therapy and natural killer (NK) cell modification for better tumor targeting, it improves immunotherapy. Additionally, CRISPR screens help overcome drug resistance by reversing mutations and studying resistance mechanisms, as well as identifying gene vulnerabilities for synthetic lethality-based therapies (Ding et al., 2023; Dekkers et al., 2020). In diseases caused by petrochemical exposure, such as those from benzene and PAHs, CRISPR-Cas9 may offers the potential to replicate mutations in models for studying disease progression and identifying drug targets. It could also correct harmful mutations in affected individuals, offering personalized treatment options (Xu, 2024; Rager et al., 2019). For instance, in a recent study, CRISPR-Cas9 technology was employed to investigate the metabolic pathways involved in retene-induced toxicity in zebrafish, a model organism for polycyclic aromatic hydrocarbon (PAH) exposure. By creating *cyp1a*-null and *cyp1b1*-null zebrafish, the researchers aimed to explore the specific roles of these enzymes in the metabolism of retene. The results revealed that *cyp1a*-null zebrafish exhibited heightened sensitivity to retene toxicity, indicating that Cyp1a plays a crucial role in detoxification. In contrast, *cyp1b1*-null zebrafish demonstrated reduced susceptibility, suggesting that Cyp1b1 may offer protective effects against toxic metabolites. The study also examined the influence of the microbiome on retene toxicity, concluding that it had little impact on the observed toxicity. Notably, zebrafish embryos were found to be most vulnerable to toxicity when exposed to retene between 24- and 48-h post-fertilization, with metabolites detected at 36 and 48 h before toxicity manifested. These findings highlight the utility of CRISPR-Cas9 in uncovering the metabolic mechanisms driving aryl hydrocarbon receptor (AhR) dependent and PAH-induced developmental toxicity (Rude et al., 2024). Similarly, the relationship between AhR and benzene was studied in another investigation. AhR plays a critical role in Th17 cell activation during the development of rheumatoid arthritis (RA). Smokers' synovial membranes show increased AhR expression due to cigarette smoke, which contains benzene and hydroxyquinone (HQ). Mice exposed to HQ exhibit worsened RA symptoms, with a higher frequency of AhR + neutrophils and Th17 cells in inflammatory tissue. However, AhR knockout mice exposed to HQ showed no signs of RA, highlighting the importance of AhR in disease progression. These findings suggest that benzene metabolites, such as HQ, exacerbate RA by activating AhR in blood cells (Scharf et al., 2020). Thus, CRISPR/Cas9 can also be widely used to correct genetic abnormalities linked to abnormal AHR expression in diseases triggered by petrochemical exposure. By precisely editing the genes involved, this technology has the potential to reverse the harmful effects of petrochemical-induced mutations. It offers a promising avenue for treating conditions where AHR dysregulation plays a key role in disease progression.

## 8 AI (artificial intelligence and radio-omics/machine learning)

Radio-omics is a sophisticated method that uses medical images to extract and analyze quantitative data. This analysis offers important insights into disease characteristics, such as tumor phenotypes, genetic profiles, and response to treatment. In cardiology, neurology, and oncology, radio-omics is widely used to improve diagnosis, prognosis, and individualized treatment plans. Through the correlation of radio-omic signatures with treatment outcomes, physicians can improve therapeutic choices, including oncology treatment response prediction. Radiomics also helps identify neurodegenerative illnesses like Alzheimer’s. It has strengthened its position in precision medicine by further enhancing biomarker identification, diagnostic accuracy, and workflow automation through its integration with AI, especially machine learning and deep learning (Maniaci et al., 2024). Artificial Intelligence (AI) plays a crucial role in risk assessment by enabling data-driven predictions and decision-making. Techniques like Artificial Neural Networks (ANN) and fuzzy logic help model complex, exposure scenarios by analyzing environmental and physiological variables. By integrating AI, risk estimation models can enhance accuracy, adaptability, and efficiency in assessing chemical exposure and health hazards. In a recent study through the inclusion of imprecise variables in health risk calculations an integrated approach was developed for evaluating benzene exposure risks in petrochemical plants. In which the suggested model dynamically estimates risk levels depending on task-specific exposure and environmental factors by combining fuzzy logic and Artificial Neural Networks (ANN). While ANN forecasts atmospheric benzene concentrations by examining wind speed, temperature, humidity, and rainfall, fuzzy logic takes into account individual factors like age, body weight, and physical activity to determine exposure levels. Because of its great adaptability, the model can be modified to incorporate expert input into fuzzy rules, providing flexibility in a range of industrial settings. This composite system provides a strong and thorough tool for assessing chemical exposure and health risks by combining several uncertainties (Novin et al., 2016). Moreover, recently another study was employed to examine the association between polycyclic aromatic hydrocarbons (PAHs) and chronic bowel disorders in U.S. adults, specifically chronic diarrhea and constipation by using data from the National Health and Nutrition Examination Survey (NHANES). Using machine learning model/unsupervised techniques such as Principal Component Analysis (PCA) and K-means clustering, researchers identified three principal components explaining 86.5% of PAH variability and finally found that higher PAH exposure was linked to an increased risk of chronic diarrhea but not constipation. Additionally, upon searching for weighted quantile sum (WQS) regression and Bayesian kernel machine regression (BKMR), further stusy confirmed this association that with specific PAHs significantly contributes to the risk of chronic diarrhea. Hence, the study highlights the potential role of environmental contaminants in bowel disorder pathogenesis and underscores the need for further research to understand underlying mechanisms and public health implications (Zang et al., 2024). Therefore, these methods have enormous potential to improve disease diagnosis and risk assessment because radiomics, along with integrated AI and machine learning models, is a rapidly developing field. By finding subtle imaging biomarkers connected to chronic diseases and environmental exposures, they could improve precision medicine if they are further developed. Deeper understanding of the relationship between harmful chemicals exposure, tissue changes, and disease progression may be possible by expanding these techniques.

## 9 Implications for regulation and policy of AI-d riven and epigenetic knowledge of petrochemical exposure

The results of the review highlight the serious health risks connected to exposure to petrochemicals, especially the way in which it can cause epigenetic changes that aid in the development of disease. The aforementioned observations underscore the pressing necessity of regulatory and policy measures intended to reduce exposure, improve risk assessment, and encourage creative treatment approaches. The need for stronger environmental controls to restrict the release of dangerous petrochemical pollutants is a crucial regulatory implication. Limits on emissions of chemicals like benzene and polycyclic aromatic hydrocarbons (PAHs) have been set by organizations like the European Chemicals Agency (ECHA) and the Environmental Protection Agency (EPA). Nonetheless, data indicates that even at low exposure levels, extended exposure may result in epigenetic changes connected to cancer and other illnesses. For instance, results from the studies together highlighted major epigenetic modification as DNA methylation which occurs due exposure to environmental chemicals, such as benzene and other and plays which may play a significant role in the development of cancer (Shimabukuro et al., 2007; Watanabe et al., 2008; Seow et al., 2012; Lei et al., 2015; Qian et al., 2019). Moreover, there are serious health risks associated with petrochemical exposure at work, especially in refineries and chemical plants. The International Labour Organization (ILO) and the Occupational Safety and Health Administration (OSHA) ought to think about updating acceptable exposure limits to include epigenetic biomarkers as early warning signs of petrochemical toxicity. Studies have shown that exposure to benzene is linked to epigenetic alterations, including histone modifications and DNA hypomethylation, which can raise the risk of leukemia and other hematologic disorders. Precision medicine strategies for at-risk groups may be advanced by incorporating these epigenetic discoveries into regulatory frameworks. To prevent petrochemical-induced mutations and aid in the creation of diagnostic instruments for early disease detection, policymakers ought to think about supporting research centered on epigenome editing. To guarantee their safe and fair use, rules must be established because these technologies present ethical and legal issues. By incorporating these epigenetic discoveries into regulatory frameworks, precision medicine strategies for vulnerable groups may be improved. Policymakers should think about providing funding for studies that use epigenome editing to prevent mutations brought on by petrochemicals and to aid in the creation of diagnostic instruments for early disease detection. But because of the ethical and legal issues these technologies bring up, rules must be put in place to guarantee their safe and fair use. In conclusion, a multi-sectoral policy approach is required in light of the growing evidence of petrochemical-induced epigenetic changes. To lessen the long-term effects of petrochemical pollution, governments, academic institutions, and industry stakeholders must work together to improve exposure guidelines, encourage environmentally friendly industrial practices, and make investments in cutting-edge health monitoring technologies (Ho et al., 2012; Li et al., 2015; Qian et al., 2019).

## 10 Future directions

To improve knowledge and reduce health risks, future studies on the epigenetic effects of petrochemical exposure should concentrate on important areas. Even though recent research has found important indicators like histone modifications and DNA methylation, more research is needed to fully understand how different epigenetic processes interact, including chromatin remodeling and non-coding RNAs. A comprehensive understanding of how petrochemical pollutants impact gene regulation and contribute to the development of disease can be obtained by integrating multi-omics techniques, such as transcriptomics, proteomics, and metabolomics. However, there are difficulties in putting multi-omics studies into practice, especially when it comes to cost, data analysis, and clinical translation. A major obstacle to conducting extensive multi-omics research is the high expense of mass spectrometry, advanced sequencing, and longitudinal cohort studies. High-throughput omics data generation necessitates costly equipment, reagents, and computer power. The ongoing collection, processing, and storage of samples for long-term studies that monitor epigenetic changes over time also raises costs. Research scope and the ability to include large, diverse populations in studies are frequently limited by these financial constraints.

Expensive multi-omics research: One of the primary barriers to large-scale multi-omics research is the high cost associated with advanced sequencing, mass spectrometry, and longitudinal cohort studies. Generating high-throughput omics data requires expensive reagents, instrumentation, and computational resources. Additionally, maintaining long-term studies that track epigenetic changes over time further increases costs due to repeated sample collection, storage, and processing. These financial constraints often limit the scope of research and the ability to include large, diverse populations in studies.

Complexity of integration and data analysis: Multi-omics studies generate vast amounts of heterogeneous data that require sophisticated computational tools for integration. Differences in data types, such as RNA-seq for transcriptomics and mass spectrometry for proteomics, make it difficult to establish direct correlations and identify biologically meaningful patterns. Additionally, variability between datasets, batch effects, and differences in experimental conditions pose challenges in ensuring reproducibility and consistency. Effective data processing demands expertise in bioinformatics, machine learning, and systems biology, which may not always be readily available in research teams (Hasin et al., 2017; Dar et al., 2023; Tariq et al., 2021).

Researchers can obtain funds through government grants, private foundations, and industry partnerships to overcome the financial obstacles. Using publicly available datasets from repositories like GEO and TCGA can minimize the need for new data collection, while cost-effective technologies like multiplex assays and nanopore sequencing can lower per-sample costs. Integrating AI and ML-based techniques into data analysis can improve predictive modeling of disease risks associated with petrochemical exposure by managing and interpreting large datasets more effectively. Batch correction techniques and multi-omics network-based models are two examples of standardized data processing pipelines that can increase study comparability and reproducibility. Furthermore, interdisciplinary partnerships among toxicologists, molecular biologists, and bio-informaticians can improve the interpretation of data. The development of non-invasive diagnostic techniques, such as liquid biopsies for the detection of epigenetic biomarkers, and close cooperation with legislators to create exposure guidelines and public health initiatives are necessary to translate research findings into clinical and policy applications. Extending population-based and longitudinal research across a range of demographics will improve precision medicine approaches to reducing the health hazards brought on by petrochemical-induced epigenetic changes. To improve our knowledge of petrochemical-induced epigenetic changes and create practical methods to lessen their negative health effects, it will be essential to address these issues using an integrated, multidisciplinary approach (Hasin et al., 2017; Dar et al., 2023; Tariq et al., 2021).

## 11 Conclusion

The growing body of research on petrochemical exposure highlights the significant role of epigenetic modifications in mediating toxicological effects and disease risks. Several investigations have demonstrated that petrochemicals, through mechanisms such as global DNA hypomethylation and the hypermethylation of tumor suppressor gene promoters, can disrupt gene function and contribute to conditions like cancer and cardiovascular disease. Alterations in specific epigenetic markers such as long interspersed nuclear element 1 (LINE1), Aluminium elements, human endogenous retrovirus (HERV), forkhead box P3 (FOXP3), and interferon gamma (IFN-γ), which are influenced by petrochemical pollutants like benzene and PAHs. These findings underline the potential of using epigenetic modifications as biomarkers for monitoring exposure and disease progression. In conclusion, epigenetic mechanisms represent heritable changes that mediate gene expression and provide critical insights into the biological impacts of environmental pollutants. The rising focus on epigenetic research in relation to petrochemicals is not only improving our understanding of exposure risks but also opening avenues for therapeutic interventions. Advances in this field are paving the way for the development of drugs capable of modulating epigenetic mechanisms, offering promising clinical responses for diseases associated with petrochemical exposure. This evolving landscape suggests that future studies should continue to investigate how these modifications can serve both as biomarkers for risk assessment and as therapeutic targets.

## Abbreviations

miRNA, microRNA; LINE1, Long interspersed nuclear element 1; HERV, Human endogenous Retro Virus; FOXP3, Forkhead Box P3; IFN-ϒ, Interferon gamma; IL-4, Interleukin –, 4; BTEX, Benzene, Toluene, Ethylbenzene, Xylene; PAH’s, Polycyclic Aromatic Hydrocarbons; Bap, Benzo(a)Pyrene; CO_2_, Carbon dioxide; CO, Carbonmonoxide; PH, Petrochemical hydrocarbons; VOC’s, Volatile Organic Compounds; HCC, Hepatocellular Carcinoma; COPD, Chronic Obstructive Pulmonary Disease; MAGE-1, Melanoma antigen –, 1; NER, Nulceotide Excision Repair; HDAC, Histone Deacetylase.
